# Identifying Contact Risks for SARS-CoV-2 Transmission to Healthcare Workers during Outbreak on COVID-19 Ward

**DOI:** 10.3201/eid2810.220266

**Published:** 2022-10

**Authors:** Marius Zeeb, Dana Weissberg, Silvana K. Rampini, Rouven Müller, Thomas Scheier, Walter Zingg, Roger D. Kouyos, Aline Wolfensberger

**Affiliations:** University Hospital Zurich and University of Zurich, Zurich, Switzerland (M. Zeeb, D. Weissberg, S.K. Rampini, R. Müller, T. Scheier, W. Zingg, R.D. Kouyos, A. Wolfensberger);; Institute of Medical Virology, Zurich, Switzerland (M. Zeeb, R.D. Kouyos)

**Keywords:** COVID-19, SARS-CoV-2, severe acute respiratory syndrome, coronavirus disease, contact tracing, disease outbreaks, healthcare personnel, infectious disease transmission, patient-to-professional, risk factors, severe acute respiratory syndrome epidemiology, severe acute respiratory syndrome transmission, healthcare delivery

## Abstract

We assessed the risk for different exposures to SARS-CoV-2 during a COVID-19 outbreak among healthcare workers on a hospital ward in late 2020. We found working with isolated COVID-19 patients did not increase the risk of COVID-19 among workers, but working shifts with presymptomatic healthcare coworkers did.

One study found SARS-CoV-2 seroprevalence to be higher among healthcare workers (HCWs) with patient contact than among those without (*1*), but another study found that HCWs were less likely to acquire SARS-CoV-2 from patients than from coworkers or someone outside the hospital (*2*). We investigated a COVID-19 outbreak in a 26-bed hospital ward with 50 HCWs in Switzerland during October–November 2020, the peak of the second COVID-19 wave. During the 43-day outbreak period, transmission chains could not be reconstructed epidemiologically or phylogenetically. Instead, we used statistical modeling to assess and compare patients and coworkers as potential sources for COVID-19 among HCWs. 

At all times, HCWs were to observe universal masking and social distancing protocols and regularly disinfect mutually used surfaces. HCWs also were to observe standard precaution measures (SPMs) for all patient contacts: wearing surgical masks at all times, eyewear when approaching a patient, and FFP2 (filtering facepiece) respirator masks during aerosol-generating procedures or prolonged contact with a patient with respiratory symptoms. For contact with patients with confirmed COVID-19, HCWs were to observe isolation precaution measures (IPMs), which, in addition to SPMs, meant wearing single-use gowns and disposing of personal protective equipment immediately after use. All patients were to wear masks when leaving bed and, starting in November 2020, when in contact with HCWs.

We assessed 3 possible risk factors as routes of exposure for HCWs: caring for contagious patients, stratified by whether using IPM or SPM when in contact with contagious patients, and working shifts during the contagious period of coworkers later found to have COVID-19. We defined the contagious period of a person with COVID-19 as the 48 hours before symptom onset, or a positive test if asymptomatic, until at least 14 days after sign/symptom onset or 2 days after signs/symptoms ended, whichever was later. HCWs were tested if symptomatic or during a staff screening on day 31 of the outbreak. 

We assumed that transmission occurred 2–10 days before symptom onset or a positive test and calculated exposure risk scores for a given day and contact type. Exposure risk scores per contact type equaled mean numbers of patient contacts when using IPM, patient contacts when using SPM, and contacts with contagious HCWs per day (Appendix Figure 1). We included all HCW workdays during the outbreak except days worked after HCWs recovered from COVID-19. To calculate hazard ratios, we used time-updated univariable and multivariable Cox proportional-hazards models with time to COVID-19 as the outcome and exposure risk scores as predictors. We also performed a sensitivity analysis for presence or absence on the ward. 

Because our analyses were part of an outbreak investigation, the Zurich Cantonal Ethics Commission waived formal ethical evaluation (Req 2021–00098). The 12 COVID-positive patients in the hospital ward were also part of a 1,118-patient study about nosocomial COVID-19 incidence in a tertiary care center (*3*). 

We found that 18/50 (38%) HCWs had COVID-19 during the study period. For the 12 patients with COVID-19 on the ward, IPM were used for 11, SPM were used for 7 of those patients until diagnosis was made; 1 patient was diagnosed only after being discharged (Table). Univariable and multivariable models indicated that COVID-19 infection among HCWs working on the ward was associated with shifts worked with coworkers subsequently found to be ill (Figure), supporting results of other studies (*4*–*6*). 

**Table:** Number of different exposures to SARS-CoV-2 for total HCW population, HCW who tested positive, and HCW who tested negative during outbreak in hospital ward, Switzerland, October–November 2020*

Type of contact	No. (%) HCWs		
	All	SARS-CoV-2–positive	SARS-CoV-2–negative
All contacts	50 (100)	18 (36)	32 (64)
Shifts with patient contact using SPM	69 (13.9)	24 (20.2)	45 (11.9)
Shifts with patient contact using IPM	143 (28.8)	31 (26.1)	112 (29.7)
Shifts with HCW contact	284 (57.3)	64 (53.8)	220 (58.4)

*IPM, isolation precaution measure; SPM, standard precaution measure; HCW, healthcare worker.

**Figure:**
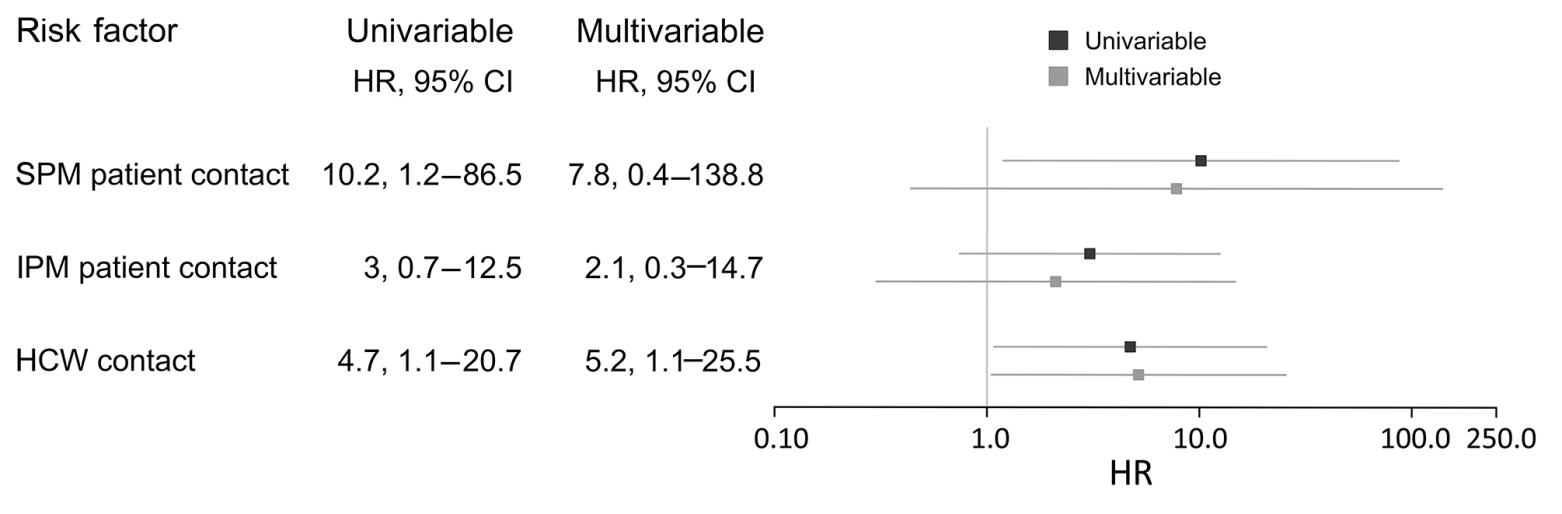
Hazard ratios and the 95% CIs for HCWs to acquire SARS-CoV-2 after using SPM and IPM for patient contact and HCW contact (i.e., contact with positive HCWs) during COVID-19 outbreak in hospital ward, Switzerland, October–November 2020. The multivariable model combined patient contact using SPM and IPM and HCW contact. HCW, healthcare worker; HR, hazard ratio; IPM, isolation precaution measures; SPM, standard precaution measures.

Our results suggested no strong association between COVID-19 in HCWs and using IPM during patient contact. Sufficiently available personal protective equipment, intensive training, and routine safety practices in handling COVID-19 patients may explain this finding. Caring for COVID-19 patients when using SPM was associated with SARS-CoV-2 infection, although only in the univariable model, pointing to a potential risk (*7*). However, we could only speculate whether our finding of increased risk resulted from the concept of SPM or as it was implemented. IPM might add extra layers of safety not only through its added protective elements but also by sensitizing HCWs to the heightened need to take precautionary measures; further investigation is needed. Ward contact, accounting for social work interactions including but not limited to those previously mentioned, showed increased SARS-CoV-2 transmission risk (Appendix Figure 2). HCWs were to wear masks, keep distance, and disinfect mutually used surfaces, but we assume full compliance at all times is unlikely. Also, social contact among peers before and after work, which might favor SARS-CoV-2 transmission, was unknown. 

Two study limitations were small sample size and lack of data from exposures outside the hospital. However, applied statistical methods enabled us to investigate and identify transmission risks. Like others (*8*), we are confident that these findings provide critical information for design and adjustment of SPM and IPM during the COVID-19 pandemic. In addition, applying our methods to larger, nonoutbreak settings might be worthwhile. More detailed weighting of specific risks taking into account distribution of incubation time (*9*) might improve estimates of transmission risk in larger studies. 

In conclusion, we provide additional evidence for SARS-CoV-2 infection risk for HCWs in contact with contagious coworkers and patients using SPM. Our findings highlight the importance of choosing protective equipment wisely and strictly adhering to safety protocols, including SPM. 

Appendix Additional information about study of contact risks for at-work SARS-CoV-2 transmission to healthcare workers, Switzerland.
