# Supplementary material for: Identifying Contact Risks for SARS-CoV-2 Transmission to Healthcare Workers during Outbreak on COVID-19 Ward
Source: Emerg Infect Dis. 2022 Oct;28(10):2134–7. doi: 10.3201/eid2810.220266 (PMC9514331; doi:10.3201/eid2810.220266)
Supplement: Appendix — Additional information about study of contact risks for at-work SARS-CoV-2 transmission to healthcare workers, Switzerland. [file 22-0266-Techapp-s1.pdf]

Article DOI: https://doi.org/10.3201/eid2810.220266

Identifying Contact Risks for SARS-CoV-2 Transmission to Healthcare Workers during Outbreak on COVID-19 Ward

Appendix

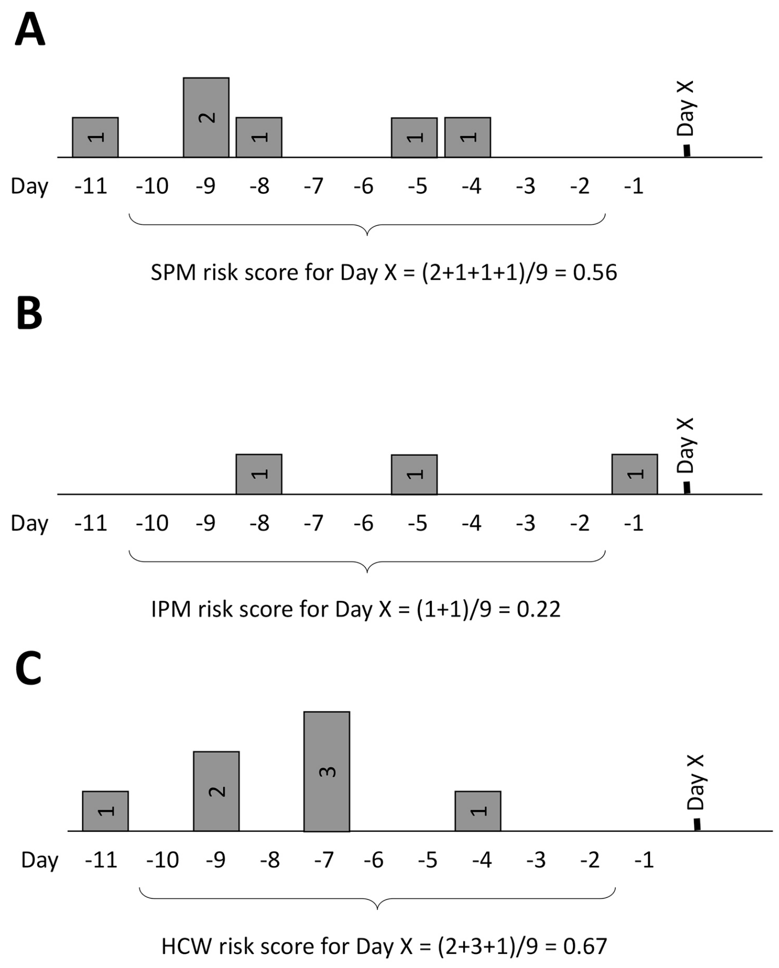

Appendix Figure 1. We assumed that transmission occurred 2–10 days before a positive test or symptom onset (Day X). Grey bars show number of contacts per day, with 1 contact defined as caring for 1 contagious patient during 1 shift or 1 shift worked with 1 contagious HCW. The risk score was then calculated as the mean contacts per day during Day X−10 to Day X−2 for each risk factor. HCW, healthcare worker; IPM, isolation precaution measures; SPM, standard precaution measures.

Risk factor	Univariable HR, 95% CI
Ward contact	13.2, 1.9-88.9

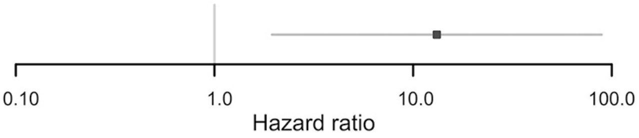

Appendix Figure 2. Hazard ratio and the 95% confidence interval for healthcare workers to acquire SARS-CoV-2 after contact on the ward. We defined ward contact as days spent working on the ward, irrespective of other contacts; we calculated risk score as mean number of workdays in the 2–10 days before a given day. HR, hazard ratio
